# Hard X-ray full-field nanoimaging using a direct photon-counting detector

**DOI:** 10.1107/S1600577522012103

**Published:** 2023-02-01

**Authors:** Silja Flenner, Johannes Hagemann, Felix Wittwer, Elena Longo, Adam Kubec, André Rothkirch, Christian David, Martin Müller, Imke Greving

**Affiliations:** a Helmholtz-Zentrum Hereon, Max-Planck-Strasse 1, 21502 Geesthacht, Germany; b Center for X-ray and Nano Science – CXNS, Deutsches Elektronen-Synchrotron – DESY, Notkestraße 85, 22607 Hamburg, Germany; c Paul Scherrer Institut, Forschungsstrasse 111, 5232 Villigen, Switzerland; Paul Scherrer Institut, Switzerland

**Keywords:** nanotomography, full-field X-ray microscopy, near-field holography, near-field ptychography, Zernike phase contrast, single-photon-counting detector, phase contrast

## Abstract

Full-field X-ray nanoimaging is a widely used tool in a broad range of scientific areas. In particular, for low-absorbing biological or medical samples, phase contrast methods have to be considered. Three well established phase contrast methods at the nanoscale are transmission X-ray microscopy with Zernike phase contrast, near-field holography and near-field ptychography. The high spatial resolution, however, often comes with the drawback of a lower signal-to-noise ratio and significantly longer scan times, compared with microimaging. In order to tackle these challenges a single-photon-counting detector has been implemented at the nanoimaging endstation of the beamline P05 at PETRA III (DESY, Hamburg) operated by Helmholtz-Zentrum Hereon. Thanks to the long sample-to-detector distance available, spatial resolutions of below 100 nm were reached in all three presented nanoimaging techniques. This work shows that a single-photon-counting detector in combination with a long sample-to-detector distance allows one to increase the time resolution for *in situ* nanoimaging, while keeping a high signal-to-noise level.

## Abstract 2

A direct photon-counting detector was used for different nanoimaging phase contrast techniques, increasing the temporal resolution.

## 1. Introduction

Hard X-ray nanoimaging and nanotomography are powerful and frequently used tools in many research areas such as materials science, biology, geology and medical science.

A classical method for full-field X-ray tomography at the nanoscale is based on a transmission X-ray microscope. It typically consists of condenser optics with a central stop focusing the incoming X-ray beam onto the sample. The image formation is achieved by an X-ray objective lens placed behind the sample, projecting the image onto the detector. However, especially for low-absorbing biological samples, contrast and dose are major issues when it comes to absorption-based X-ray imaging. High X-ray energies allow for a lower total dose, but require phase contrast methods to visualize low-absorbing specimens at high spatial resolution. For a transmission X-ray microscope, the method of choice is Zernike phase contrast (ZPC) (Zernike, 1942 ▸; Schmahl *et al.*, 1994 ▸) which can easily be implemented into an existing transmission X-ray microscope by inserting a phase ring in the back-focal plane of the objective lens. The advantage of ZPC is that the contrast of the image is directly enhanced so no phase retrieval step is necessary.

In a transmission X-ray microscope, the resolution is limited by the numerical aperture (NA) of both the condenser optics NA_con_ and objective lens NA_obj_. In the case of a similar NA, the resolution is improved by a factor of two: *d* = 1.22λ/(NA_con_ + NA_obj_) = 0.61λ/NA. For a transmission X-ray microscope based on Fresnel zone plates (FZPs), the resolution is simply limited by the outermost zone width *d*_*r*_ of the FZP and beamshaping condenser: *d* = 0.61*d*_*r*_ (Attwood, 2007 ▸; Born *et al.*, 1999 ▸).

Near-field holography (NFH) (Cloetens *et al.*, 1999 ▸) is another phase contrast method. In combination with nano-focusing optics it allows imaging at the nanoscale, offering high flexibility with a scalable field of view (FOV) and magnification, quantitative phase retrieval and a large working distance. The latter allows one to integrate sample environments, *e.g.* for *in situ* experiments, more easily. In NFH, the magnification is achieved by placing the sample in the divergent beam of the focusing optics. In this case, the contrast in the measured hologram is formed by propagation of the wavefield behind the object in free space. In the NFH setup, no optics are placed behind the sample. Such optics usually have a limited efficiency and can introduce further artifacts (*e.g.* aberrations). Therefore, NFH is also referred to as a lens-less imaging scheme which makes NFH also very dose efficient. The resolution of NFH is limited by the focal spot size.

Near-field ptychography (NFP) (Stockmar *et al.*, 2013 ▸; Xu *et al.*, 2020 ▸) offers the opportunity to increase the FOV compared with plain NFH and to obtain a reconstruction without prior sample knowledge, also for non-uniform illuminations. For NFP, holographic projections are recorded at different lateral sample positions. With a large and sensitive detector the scattering information outside the primary beam area can be exploited. Thus, the resolution is no longer limited by the focal spot size, but by the achievable numerical aperture of the detector.

Especially at synchrotron radiation sources these full-field methods offer fast nanotomography for both transmission X-ray microscopy (TXM) (Ge *et al.*, 2018 ▸; Flenner *et al.*, 2020*c* ▸) and NFH (Villanova *et al.*, 2017 ▸). From the point of view of data analysis, the noise level of a detector, *i.e.* the contrast-to-noise ratio (CNR), is very important, especially if a high time resolution is required.

Two types of detector chips are commonly in use: charge-coupled device (CCD) systems usually have a lower noise level, but suffer from long readout and dead-times, making them less suitable for fast tomography. Complementary metal-oxide-semiconductor (CMOS)-based cameras on the other hand offer a much faster readout and small pixel sizes and are therefore well suited for fast nanoimaging approaches (Allé *et al.*, 2016 ▸; Flenner *et al.*, 2020*c* ▸). Often a detector system is used where the scintillator is coupled to a light optical system, which magnifies the image onto the camera chip. Hence, many photons are lost in the complex system for light optical magnification which increases the noise level significantly. Also systems without a light optical magnification are in use, *e.g.* the Hamamatsu CMOS (C12849), where the scintillator is directly coupled to the chip via a fiber optical plate. Here, a long sample-to-detector distance is needed to achieve a sufficient magnification in the X-ray regime due to the small divergence of the focused X-ray beam. This makes the system more efficient, but the noise level, mainly a combination of detector readout noise and photon noise, is still not negligible (Flenner *et al.*, 2022 ▸).

One approach to improve the image quality in full-field imaging techniques is the use of single-photon-counting pixel detectors (Manolopoulos *et al.*, 1999 ▸; Ponchut *et al.*, 2021 ▸). Such detectors provide a noiseless readout and can threshold photons within a specific energy range, making them highly attractive for fast tomography approaches. In addition, they offer an almost single-pixel point spread function (PSF) (Donath *et al.*, 2013 ▸). In comparison, CMOS cameras have a PSF of 2 to 3 pixels, limiting the spatial resolution. Among the commercially available single-photon-counting detectors, the X-Spectrum LAMBDA detector (Pennicard *et al.*, 2013 ▸, 2014*a* ▸,*b* ▸) based on the MEDIPIX3 sensor (Ballabriga *et al.*, 2013 ▸; Manolopoulos *et al.*, 1999 ▸) currently offers the smallest pixel size (55 µm) and has been used for imaging techniques such as far-field ptychography (Wilke *et al.*, 2014 ▸). Other single-photon-counting detectors with larger pixel size are also used for imaging, *e.g.* the Dectris Eiger with a pixel size of 75 µm (Dinapoli *et al.*, 2011 ▸; Schropp *et al.*, 2020 ▸). In addition, photon-counting detectors are frequently used in benchtop devices and for medical purposes to reduce dose and scan time (Ballabriga *et al.*, 2021 ▸). However, the physical pixel sizes of commercially available detectors are currently limited to >50 µm (Ballabriga *et al.*, 2021 ▸). Therefore, full-field nano­imaging applications in the hard X-ray regime using photon-counting detectors require a much stronger demagnification of the pixel size compared with CMOS cameras with much smaller pixels in the range of 5 to 10 µm. This demagnification can for example be achieved via a large sample-to-detector distance.

At the imaging beamline P05 at PETRA III (DESY, Hamburg), a sample-to-detector distance of up to 22 m is accessible. In this article, TXM measurements using a direct photon-counting detector are shown and compared with the performance of a conventional CMOS system. In addition, NFH and NFP are presented and evaluated using a LAMBDA detector.

## 2. Materials and methods

**Figure 1:**
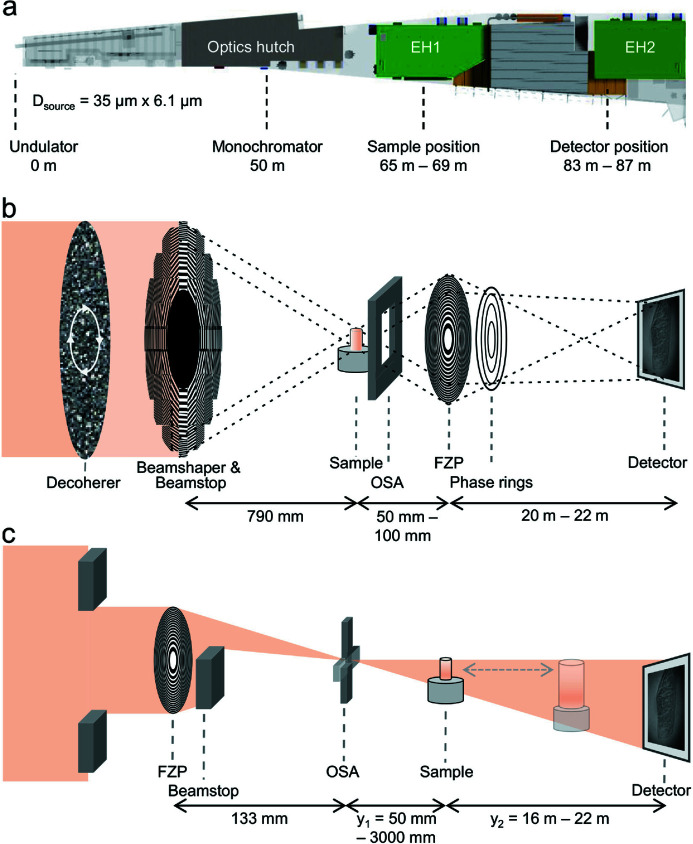
(*a*) Overview of the beamline P05 with relevant hardware components and their distances to the source. The nanotomography setup is located in the first experimental hutch (EH1) while the camera is placed in the second experimental hutch (EH2). (*b*) Zernike phase contrast implemented in a standard transmission X-ray microscope and (*c*) near-field holography setup using a Fresnel zone plate (FZP) as focusing optics. This setup was also used for the near-field pytchography measurements.

The experiments were performed at the P05 nanotomography endstation which is operated by the Helmholtz-Zentrum Hereon at the storage ring PETRA III at DESY, Hamburg, Germany (Ogurreck *et al.*, 2013 ▸; Greving *et al.*, 2017 ▸, 2018 ▸). The nanotomography endstation is located at the first experimental hutch (EH1) of the imaging beamline P05 [Fig. 1 ▸(*a*)]. The beamline also hosts a microtomography experiment located in a second experimental hutch (EH2). Since only one experiment can run at the same time, the detector can be placed in EH2, enabling a sample-to-detector distance of up to 22 m [Fig. 1 ▸(*a*)]. This allows for a high magnification in the X-ray regime, enabling nanoimaging without additional light optical magnification. The different imaging techniques were performed at a photon energy of 11 keV, monochromatized by a channel-cut Si-111 monochromator. A Si-based LAMBDA 750k single-photon-counting detector with a pixel size of 55 µm × 55 µm was used (Pennicard *et al.*, 2013 ▸, 2014*a* ▸,*b* ▸). The detector area consists of 12 individual chips of 256 × 256 pixels, arranged on a 2 × 6 grid. At the edges between adjacent chips there are larger pixels of size 55 µm × 165 µm (edges) and 165 µm × 165 µm (corners). These are split into three pixels inside the detector software, resulting in a final image size of 1556 × 516 pixels. The LAMBDA was used in 24 bit mode (16.7 million photons per pixel) which enables a maximum frame rate of 1000 frames per second (full frames). For comparison, an X-ray sCMOS camera (Hamamatsu C12849-101U, 6.5 µm pixel size, 2048 × 2048 pixels, 16 bit image depth) with a 10 µm Gadox scintillator was used. The Hamamatsu’s dynamic range is reduced due to readout noise to a range of 18000:1 at a frame rate of 30 frames per second (full frames).

### 2.1. Zernike phase contrast setup

The TXM setup for ZPC is shown in Fig. 1 ▸(*b*). The beamshaping condenser (1.8 mm diameter) (Vartiainen *et al.*, 2014 ▸) and the beamstop (800 µm diameter) create a hollow cone illumination and focus the beam on a 50 µm × 50 µm spot in the sample plane. Order sorting apertures (OSA) block the higher diffraction orders of the beamshaper. An FZP is placed behind the sample and serves as an objective lens. Depending on the desired FOV and magnification, FZPs with different diameters and outermost zone width *d*_*r*_ can be chosen. A rotating piece of paper is mounted in front of the microscope in order to decrease the impact of disturbing phase effects originating from the high coherence of the beam. For ZPC, phase rings matching the aperture of the FZP and the beamshaper are placed in the back-focal plane of the objective lens. The detector is mounted in the adjacent experimental hutch at a distance of 20.7 m, utilizing the high magnification in the X-ray regime. At this distance, the image created by the FZP has a size of approximately 14 mm × 14 mm and therefore covers the whole detector chip of the Hamamatsu detector or a single chip of the LAMBDA detector. A FOV of approximately 256 × 256 pixels on the LAMBDA is projected on a single chip. When placed in the FOV, the large pixels between the chips could induce ring-like artifacts in the tomographic reconstruction. Details of the different configurations for TXM are given in Table 1 ▸.

### 2.2. Near-field holography setup and ptychographic measurements

The NFH setup at the P05 nanoimaging endstation is based on a single FZP [Fig. 1 ▸(*c*)] (Flenner *et al.*, 2020*a* ▸). The FZP with a diameter of 300 µm and outermost zone width of *d*_*r*_ = 50 nm focuses the beam to a small spot of 1.22*d*_*r*_ = 61 nm which limits the achievable resolution. A beamstop is placed behind the FZP, covering half of the FZP and blocking the direct beam. In the focal distance of 133 mm, OSAs block the higher diffraction orders of the FZP. For the holotomographic measurements, the sample was placed at a defocus distance *y*_1_ depending on the desired FOV and magnification. In the case of a cone beam setup the magnification *M* is given by *M* = *y*_2_/*y*_1_, where *y*_2_ is the detector distance [Fig. 1 ▸(*c*)]. Thus with *y*_1_ the magnification or FOV can be adjusted to the needs of the experiment. More details of the measurements can be found in Table 2 ▸.

For the NFP measurements, we kept the same setup as for NFH. The samples were placed at a defocus distance of 36.8 mm and scanned across the beam in a 6 × 6 raster. The step size was 1 µm and each point was randomly offset horizontally and vertically by up to 1 µm to avoid scan grid artifacts. Each image was exposed for 1 s. The images from the LAMBDA were padded to a size of 1024 × 1024 pixels. Because the detector panel of the LAMBDA is only 516 pixels high, the parts of the image outside the detector area were filled with zeros. Further details of the measurements are given in Table 3 ▸. In ptychographic imaging, a structured illumination can improve the reconstruction (Stockmar *et al.*, 2013 ▸). To this end, for the scan of the spider hair, a piece of paper was inserted in front of the FZP to add diversity into the illuminating wavefront.

### 2.3. Data processing

The three different phase contrast methods require slightly different processing protocols. This includes a phase retrieval step in the case of NFH and NFP, while the ZPC data, after the flat-field correction, can be directly processed to obtain the tomographic reconstruction. For NFH, the phase reconstructions were obtained from a single distance measurement by an iterative alternating projections algorithm based on the work of Wittwer *et al.* (2022 ▸), integrated in the *HoloTomoToolbox* (Lohse *et al.*, 2020 ▸). Prior to the reconstruction, the projections have been corrected with a dynamic flat-field correction based on a principal components analysis (PCA) (Van Nieuwenhove *et al.*, 2015 ▸; Hagemann *et al.*, 2021 ▸). For NFP, the phase retrieval was carried out via the refractive ptychographic iterative engine (refPIE) (Wittwer *et al.*, 2022 ▸), using the Fresnel propagator instead of the usual Fraunhofer propagator. Due to the required precision of the sample positioning system used for scanning, a position refinement was needed in the reconstruction process (Schropp *et al.*, 2013 ▸). We accelerated the reconstruction by using Nesterov-accelerated gradients (Maiden *et al.*, 2017 ▸).

The tomographic reconstructions of the TXM and holography scans were obtained using the gridrec algorithm (Dowd *et al.*, 1999 ▸) with a Shepp–Logan filter implemented in *tomopy* (Gürsoy *et al.*, 2014 ▸).

The CNR of the TXM tomograms was calculated by the following equation (Muhogora *et al.*, 2008 ▸), 

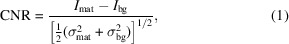

where *I*_mat_ and *I*_bg_ are the mean gray values in the material and the background, and σ_mat_ and σ_bg_ are the standard deviations for these regions. The ratio is calculated on the unfiltered TXM slices, using the mask of the segmented volume.

The resolution was estimated via the Fourier ring correlation (FRC, 2D) and Fourier shell correlation (FSC, 3D). For the tomograms, the projections were divided in two stacks and the corresponding reconstructions were then used as an input for the FSC. For the 2D phase reconstructions, several holograms were taken with the same exposure time. For NFP, the scans were split into two halves and reconstructed separately. The half-period resolution was calculated from the FRC/FSC curves using the half-bit threshold criterion (Van Heel & Schatz, 2005 ▸).

### 2.4. Samples

Two different samples were used. The first one, a low-absorbing biological sample, was a spider attachment hair (Roscoe & Walker, 1991 ▸; Niederegger & Gorb, 2006 ▸; Schaber *et al.*, 2019 ▸; Flenner *et al.*, 2020*b* ▸). These approximately 10 µm-wide and up to 1 mm-long hairs enable spiders to walk upside down on many surfaces. The hairs are built up hierarchically: a single attachment hair located at the feet of the spider is covered by several hundreds of smaller hairs (microtrichia) with diameters of 150 nm to 300 nm, ending in spatula-shaped adhesive tips. These fine elements make the spider attachment hair an ideal test sample for phase contrast nanoimaging.

The second, larger and more complex sample was a tardigrade. These species are able to survive under extreme conditions and are approximately 50 µm to 150 µm in diameter and up to 800 µm long (Gross *et al.*, 2019 ▸). The tardigrade is therefore well suited as a test sample for tomography with a large FOV and region of interest (ROI).

## 3. Results

In the following, a comprehensive comparison of the three methods, ZPC, NFH and NFP, is given.

### 3.1. Zernike phase contrast

**Figure 2:**
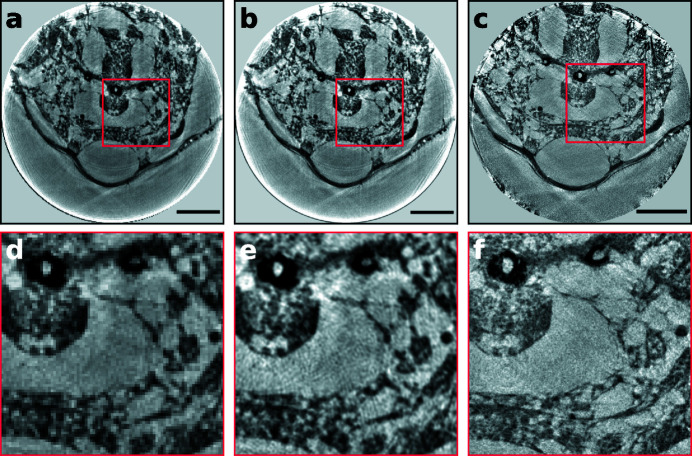
Reconstructed slices of the same tardigrade specimen imaged with Zernike phase contrast and the same optics but recorded with two different detectors. (*a*) Recorded with the LAMBDA. (*b*) Projections recorded with the LAMBDA and upsampled by a factor of four prior to the tomographic reconstruction. (*c*) Tomographic slice acquired with the Hamamatsu camera. Panels (*d*)–(*f*) show the magnified ROI indicated by the red boxes in (*a*)–(*c*). Note that the sample has been removed from the rotation axis between the two scans and may not show the exact same region. No filtering or further post-processing applied. The scale bar indicates 10 µm.

For the first time, a photon-counting detector was used for a transmission X-ray microscope at a synchrotron radiation source. Fig. 2 ▸ shows reconstructed slices of a tardigrade derived from data taken with two different detector types with exactly the same experimental setup, *i.e.* distances and optics. Here, optics with 50 nm outermost zone width and an FZP with a diameter of 150 µm were used. More details of the measurements can be found in Table 1 ▸. Figs. 2 ▸(*a*) and 2 ▸(*b*) were recorded with the LAMBDA detector, while Fig. 2 ▸(*c*) was acquired with the Hamamatsu camera. A magnified image of the ROI, indicated by the red boxes, is shown in Figs. 2 ▸(*d*)–2 ▸(*f*). Both scans were recorded with the same number of projections (1600) but different exposure times of 0.5 s (Hamamatsu) and 0.2 s (LAMBDA) due to the lower intrinsic noise of the LAMBDA detector.

The effective pixel size reached with this setup was 21.4 nm for the Hamamatsu detector and 181 nm for the LAMBDA photon-counting detector; this corresponds to the ratio of the physical pixel sizes (6.5 µm for the Hamamatsu and 55.0 µm for the LAMBDA). Covering the full detector chip of the Hamamatsu (2048 × 2048 pixels) and a single chip on the LAMBDA (256 × 256 pixels) yields a comparable FOV – 44 µm and 46 µm, respectively. Due to a PSF of 2 to 3 pixels of the Hamamatsu detector, the images are usually binned in order to increase the CNR and decrease the storage space, without losing spatial resolution. At the same time, the LAMBDA data can be upsampled by a factor of four [Figs. 2 ▸(*b*), 2 ▸(*e*)], yielding a comparable effective pixel size of 45.3 nm. Upsampling of the projections prior to the reconstruction yields additional details, as the tomography scan was heavily oversampled, and features can be recognized more easily (Dudak *et al.*, 2017 ▸). The image contrast is higher in the LAMBDA scan, whereas the resolution is still higher in the Hamamatsu scan: the effective pixel size for the LAMBDA (181 nm) is almost a factor of two larger compared with the half-period resolution calculated from FRC for the sCMOS camera (98 nm).

The limiting factor for achieving a higher spatial resolution is currently the large physical pixel size of single-photon-counting detectors. Three different approaches to overcome this limitation will be discussed in the following: (i) decreasing the radius *r* of the FZP, (ii) decreasing the outermost zone width *d*_*r*_ of the FZP, and (iii) increasing the FZP-to-detector distance *y*. All three approaches result in a higher magnification in the X-ray regime, which can be calculated by 

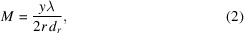

where λ is the wavelength of the X-rays. For typical values, refer to Table 1 ▸.

The first option (i) is the utilization of an objective FZP with a smaller diameter while keeping the same *d*_*r*_, *i.e.* NA. In order to achieve a resolution comparable with that of the sCMOS detector, a diameter of 83 µm is required. One disadvantage of this approach, however, is that the focal distance is reduced, limiting the space between the objective lens and the sample, hindering the implementation of larger *in situ* environments.

The second option (ii) to increase the X-ray magnification is to reduce the outermost zone width of the optics, thereby increasing the NA. Here, also the theoretical resolution can be pushed further from 30.5 nm to 18.3 nm, as the resolution limit *d* of TXM is determined by the outermost zone width *d*_*r*_ of the optics (*d* = 0.61*d*_*r*_). However, also here the focal distance is drastically reduced. In addition, the focal depth is also reduced, *e.g.* from 44 µm (*d*_*r*_ = 50 nm) to only 16 µm (*d*_*r*_ = 30 nm). For tomography, a focal depth smaller than the FOV is problematic since the entire sample needs to be in focus for the image to be considered as a simple projection of the specimen. From the fabrication side, the achievable aspect ratio of the FZP structures is limited. This means that the structure height for FZPs with smaller outermost zone width is lower, resulting in a less efficient focusing and thus less flux on the detector. To achieve an effective pixel size of 98 nm with this approach, an outermost zone width of 13 nm would be required, which is currently not feasible from the fabrication side.

The third approach (iii) to gain a higher magnification with ZPC is to simply increase the distance from the sample to the detector. For example, to achieve an effective pixel size of 98 nm with the LAMBDA, a distance of 36 m is needed. Such large sample-to-detector distances are currently not feasible at P05 or, to our knowledge, at any other nanoimaging beamline.

**Figure 3:**
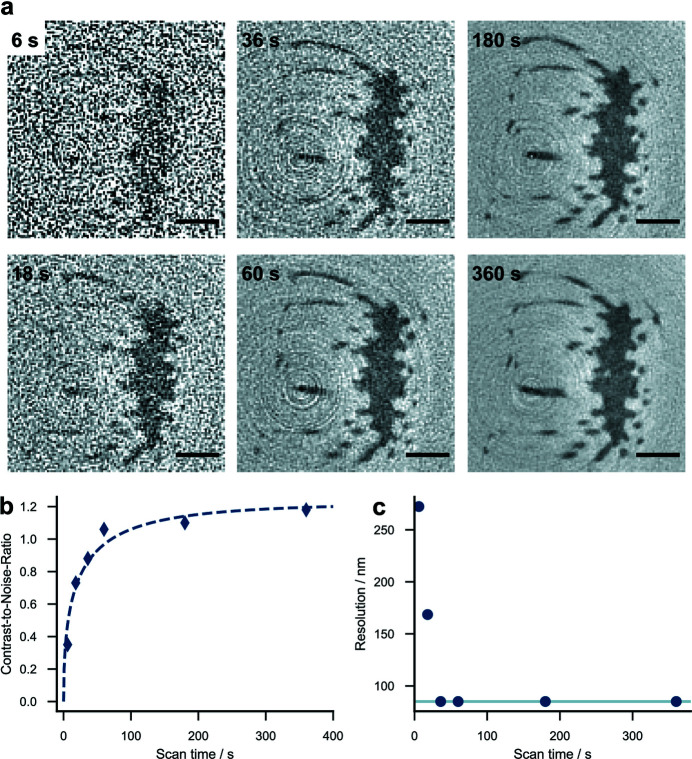
Fast Zernike phase contrast tomography with a photon-counting detector. (*a*) Reconstructed slices of a spider attachment hair with different scan times. (*b*) CNR for the different scan times. For details of the fit, refer to Flenner *et al.* (2020*c* ▸). A high CNR is already reached after 60 s. (*c*) Half-period resolution as calculated from the FRC. The effective pixel size (85 nm, solid horizontal line) limits the resolution after 36 s scan time. The scale bar indicates 2 µm.

In order to achieve a high spatial resolution with the LAMBDA, we combined the approaches of (i) and (ii) and used an FZP with smaller diameter and smaller outermost zone width. Since not all arbitrary sizes and outermost zone widths of FZPs were available, an FZP with *d*_*r*_ = 30 nm and a diameter of 120 µm was chosen, yielding an effective pixel size of 85 nm. Although the effective pixel size is still much larger than for the Hamamatsu detector and only a single chip is used (256 × 256 pixels), the resolution turned out to be sufficient to resolve the fine microtrichia in the spider attachment hair [Fig. 3 ▸(*a*)]. High-contrast tomograms with low noise can be acquired much faster, even with the lower efficiency of the 30 nm optics compared with the 50 nm optics. Fig. 3 ▸(*a*) shows the reconstructed slices of a spider attachment hair for different scan times, from 6 s up to 6 min. Details of the parameters of the scan series can be found in Table 4 ▸. With an extent of 100 pixels, the spider hair requires approximately 314 projections for a fully sampled tomogram. Apart from the 6 s scan, all scans fulfill the Crowther criterion. In the very short scans (6 and 18 s) the reconstructions suffer from a high noise level. However, already in the 36 s scan, single microtrichia are clearly resolved. The noise in this scan could be easily removed using, for example, an iterative non-local means filter (Bruns *et al.*, 2017 ▸) or a machine learning based approach (Pelt & Sethian, 2018 ▸; Hendriksen *et al.*, 2020 ▸; Flenner *et al.*, 2022 ▸). The slices obtained from scans with longer acquisition time show a decreasing noise level and a high CNR [Fig. 3 ▸(*b*)]. The CNR increases only slightly after 60 s total scan time. At this scan time, 423 photons per pixel were measured in a single projection on average, corresponding to 38.9 × 10^4^ photons per pixel in the full tomogram (Table 1 ▸). After 36 s scan time (11.5 × 10^4^ photons per pixel for a tomogram), the FSC curves show correlations up to the highest sampled frequency, indicating that the resolution is now limited by the effective pixel size rather than by noise [Fig. 3 ▸(*c*)]. However, due to limited sampling, meaning the effective pixel size, the interpretation of FSC curves at these high frequencies is ambiguous (Van Heel & Schatz, 2005 ▸). A scan time longer than 360 s is therefore not recommended, as it increases the risk of additional sample movements and therefore artifacts in the reconstruction. The CNR maximum for the Hamamatsu detector is reached only after 15 to 30 min using significantly more efficient optics (Flenner *et al.*, 2020*c* ▸). For comparison: the tardigrade in Fig. 2 ▸ was measured in 360 s with 3540 photons per pixel (5.66 × 10^6^ photons per pixel for a tomogram). This high number of photons was achieved due to much more efficient optics. Thus, the scan time could have been reduced by a factor of roughly 15.

**Figure 4:**
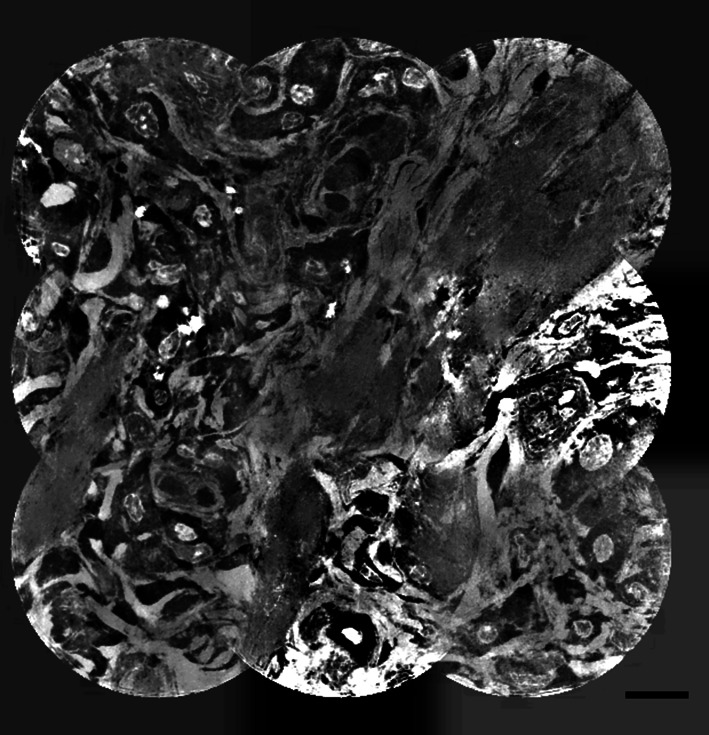
Slice of a stitched volume of a rat lung. The volume was obtained from 27 individual scans with a step size of 35 µm. The scale bar indicates 10 µm.

In conclusion, the scan time can be significantly reduced compared with the sCMOS detector, thanks to the photon-counting detector. This also enables the stitching of sub-tomograms to larger volumes of specimens such as biological tissue (Longo *et al.*, 2020 ▸; Töpperwien *et al.*, 2019 ▸). Fig. 4 ▸ shows a slice of a large volume of a rat lung. For details of sample preparation, refer to Longo *et al.* (2020 ▸). The tomogram shows a volume of 105 µm × 105 µm × 105 µm obtained from 3 × 3 × 3 = 27 individual tomography scans with an effective pixel size of 181 nm and a step size of 35 µm.

The scans were individually reconstructed using the above-described procedure and stitched with the 3D stitching plugin (Preibisch *et al.*, 2009 ▸) available in *FIJI* (Schindelin *et al.*, 2012 ▸). This enables a high-resolution reconstruction of relevant tissue volumes at short acquisition times while at the same time being non-destructive without any requirements of staining or slicing. The obtained 3D volumes can be used to complement classical 2D histology, opening the door for correlative imaging techniques (Töpperwien *et al.*, 2020 ▸; Albers *et al.*, 2021 ▸).

### 3.2. Near-field holography

**Figure 5:**
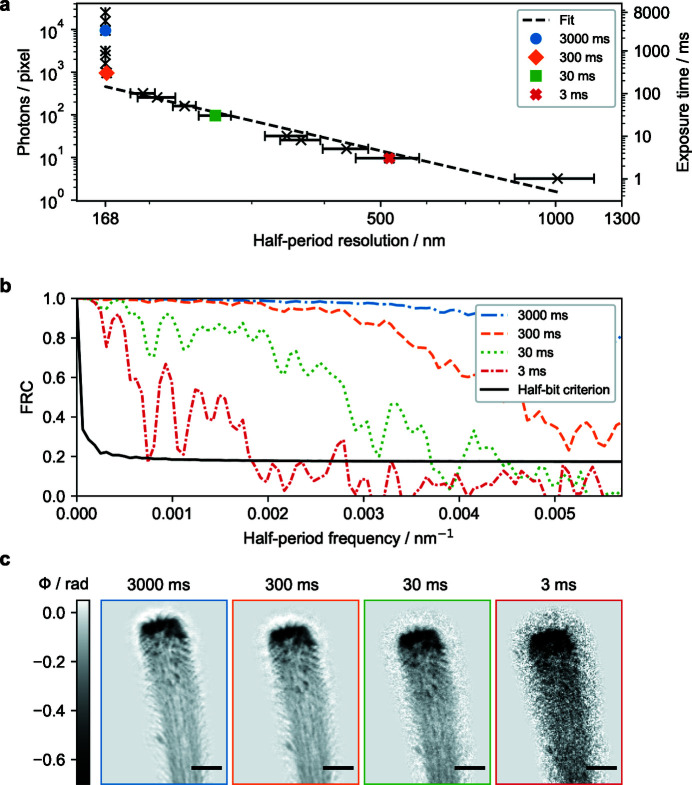
(*a*) Half-period resolution of holographic reconstructions (2D projections) as a function of the number of photons per pixel. (*b*) Estimation of the half-period resolution via the FRC for different exposure times. (*c*) Phase reconstructions of a spider attachment hair for different exposure times ranging from 3 to 3000 ms. At 300 ms, the detector sampling limits the achievable resolution. The scale bar indicates 5 µm.

Near-field holotomography offers quantitative imaging and at the same time the dose is significantly reduced compared with TXM as no optics are placed behind the sample. A single hologram is sensitive to noise, since the finest fringes, which determine the resolution, need to be sampled with a sufficiently high CNR for the phase reconstruction. Therefore, the resolution has a strong relation to the number of photons, *i.e.* dose on the sample (Rudolph *et al.*, 1990 ▸; Kirz *et al.*, 1995 ▸; Howells *et al.*, 2009 ▸; Hagemann & Salditt, 2017 ▸; Du *et al.*, 2020 ▸). Using a single-photon-counting detector allows for testing the theoretical fluence–resolution predictions, *i.e.* the number of photons per pixel needed to achieve a certain resolution in an experiment. We recorded multiple flat fields and holograms of an object at different exposure times in the range from 3 ms to 8000 ms. The holograms were flat-field corrected using the PCA method (Van Nieuwenhove *et al.*, 2015 ▸; Hagemann *et al.*, 2021 ▸), where the PCA was computed separately for each exposure time. Note that the division of the two noisy images is a situation which in general is not treated in today’s existing theoretical studies. The reconstructions were obtained from the flat-field corrected holograms with an iterative projection algorithm (Wittwer *et al.*, 2022 ▸; Hagemann *et al.*, 2021 ▸) using a self-refining support and constraints for the physically allowed values for absorption and phase shifts. The low-absorbing spider hair can be handled as a pure phase object so the allowed values for the absorption were set to zero. The measured 2D resolution as a function of number of photons per pixel is displayed in Fig. 5 ▸(*a*). The number of photons per pixel was calculated by averaging over a series of flat fields for each exposure time. The half-period resolution was calculated on 30 reconstructed projections via the FRC for each exposure time [Fig. 5 ▸(*b*)]. Again, we observe correlations up to the highest sampled frequencies.

From the fit of the data we observe a scaling of the resolution *d* as a function of the number of photons per pixel proportional to *d* ^−3.2±0.3^. This trend continues even at the highest sampled frequencies close to the pixel size limit. This value is well between calculations of the scaling from coherent (*d* ^−4^) (Starodub *et al.*, 2008 ▸) and incoherent (*d* ^−3^) (Shen *et al.*, 2004 ▸) cases. Note that the current magnitude projection used in the algorithm is implemented in a straightforward manner and does not take the noise present in the measurements into account. Because of too sparse sampling of the exposure times, the number of photons necessary to achieve the best (detector limited) resolution is determined from the fit to be 460 photons per pixel, corresponding to an exposure time of 150 ms.

Exemplary reconstructions for selected exposure times are shown in Fig. 5 ▸(*c*). Even for very short exposure times of only 3 ms, the outlines of the spider hair can be identified by eye [Fig. 5 ▸(*c*)]. Single microtrichia can already be resolved at lower spatial resolution with an exposure time of 30 ms.

**Figure 6:**
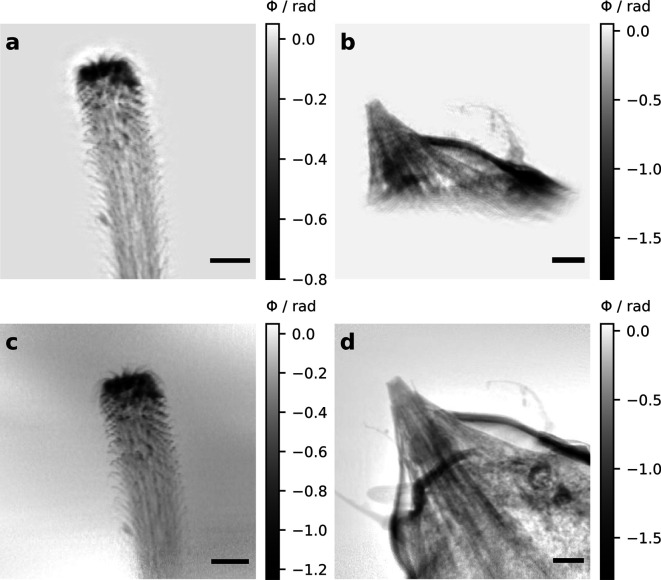
Phase reconstructed projections of two test samples, a spider attachment hair [(*a*), (*c*)] and a tardigrade head [(*b*), (*d*)], obtained with different phase contrast methods using a LAMBDA detector. (*a*), (*b*) Near-field holography and (*c*), (*d*) near-field ptychography. Note: (*b*) and (*d*) might not show exactly the same angle and region, since the sample was remounted between the two experiments. The scale bar indicates 5 µm.

The higher sensitivity of the LAMBDA detector allows one to significantly reduce the exposure time needed for a single hologram, compared with a sCMOS camera (Flenner *et al.*, 2020*a* ▸). Two test samples were measured at different magnifications, and details of the measurements can be found in Table 2 ▸. The phase reconstructions of the test samples are shown in Figs. 6 ▸(*a*) and 6 ▸(*b*). An effective pixel size of 168 nm was obtained for the spider hair at a defocus distance of 62.8 mm. Due to the small PSF of the LAMBDA, the microtrichia with diameters between 150 and 300 nm can clearly be resolved [Fig. 6 ▸(*a*)]. Since the tardigrade is not a pure phase object, the phase reconstruction is more challenging. Here, an effective pixel size of 98 nm was achieved. The phase could be reconstructed from only one distance, *i.e.* with a single exposure, revealing the inner structures of the tardigrade’s head [Fig. 6 ▸(*b*)]. In conclusion, NFH in combination with photon-counting detectors offers a fast and low-dose imaging method, especially well suited for low-absorbing biological specimens.

### 3.3. Near-field ptychography

NFP (Stockmar *et al.*, 2013 ▸, 2015 ▸; Xu *et al.*, 2020 ▸) offers two advantages over NFH: (i) the FOV is no longer limited by the beam size, because the sample is scanned transversally to the X-ray beam; (ii) ptychography is able to separate the illuminating wavefront from the transmission of the sample and therefore does not require flat fields and can be used even for non-planar wavefronts (Maiden & Rodenburg, 2009 ▸).

As the spider hair is smaller than the FOV and only weakly phase shifting, the NFP reconstruction is similar to the NFH result, see Fig. 6 ▸(*c*). Considering also the longer scan time due to the stepping of the sample and therefore also higher dose, NFH is more suited for such small samples.

In contrast, the reconstruction of the tardigrade sample highlights the strength of NFP [Fig. 6 ▸(*d*)]. The FOV is larger and every part of the head is reconstructed with more fidelity than in NFH. While the NFH reconstruction requires manual tuning of phase and absorption constraints, NFP can reconstruct the tardigrade without prior knowledge of the sample. The calculated resolution is similar for both methods.

The strength of the ptychographic measurements is that both object and illumination function are reconstructed. This reduces the influence of a non-uniform illumination on the sample reconstruction caused by a non-ideal flat-field correction. In ptychography, it is not only the diffraction inside the direct beam that contributes to the reconstruction, but also the scattering signal around it. Typically, the direct beam is three orders of magnitude more intense than the scattering around it. Here, a photon-counting detector shows its full potential due to its high dynamic range and single-photon sensitivity.

Currently, the instrument at P05 is not optimized for ptychographic imaging in terms of motor movement and accessible scattering angle. The detector aperture is slightly larger than the direct beam and most of the scattered photons are blocked due to the limited diameter of the beam pipes, which connect the two experimental hutches. A larger pipe and detector aperture would allow one to extend the FOV and improve the resolution due to the accessibility of larger scattering vectors.

## 4. Conclusion and outlook

We presented a transmission X-ray microscope setup using a single-photon-counting detector. The combination of a large sample-to-detector distance with the LAMBDA detector allows one to realize small effective pixel sizes, significantly reduced scanning times for tomography and at the same time a high contrast-to-noise ratio. The reduced scan time leads to less dose on the sample and less drift during the measurement.

In NFH, extremely short exposure times are possible. While the outlines of the samples are already visible at 3 ms, a phase reconstruction of a projection with reasonable quality can be obtained at only 30 ms exposure time. At exposure times of 150 ms, the resolution is no longer limited by photon noise, but by the sampling of the detector (effective pixel size).

NFP shows its potential especially for large samples with higher absorption and a strong phase shift. Here, the reconstruction quality can be improved and the FOV can be enlarged compared with NFH, while the spatial resolution is similar, as also stated by Monaco *et al.* (2022 ▸).

A new detector aiming to break the pixel size limit of 50 µm is currently under development (Dinapoli *et al.*, 2014 ▸; Ramilli *et al.*, 2017 ▸). In addition to the small pixel size of only 25 µm, it offers sub-pixel interpolation. In the future, such a detector has great potential to improve the achievable spatial resolution, especially in TXM mode, if readout speed is drastically improved.

The presented setups have been developed at the third-generation storage ring PETRA III. Meanwhile, the first fourth-generation synchrotron radiation sources have started operation, *e.g.* MAX IV and ESRF-EBS. An upgrade is also planned for PETRA III aiming towards a diffraction-limited storage ring. The new technical developments allow for a significant reduction of the emittance in the horizontal direction, increasing the spectral brightness by one to two orders of magnitude (Schroer *et al.*, 2018 ▸). The higher coherent flux at fourth-generation sources further increases the time resolution for the imaging modalities presented in this work. Although direct converting detectors without the use of light optical systems offer the great advantage of efficient X-ray detection, there is an issue which cannot be solved easily: the FOV that can be captured with the detector is limited by the achievable numerical aperture in the hard X-ray regime and hence by the X-ray optics and the microscope geometry. Therefore, a large sample-to-detector distance is a key factor in pushing the spatial resolution even further. In particular, the presented holotomography setup will benefit from the higher coherence at a fourth-generation source: the focal spot size of an FZP is influenced by the coherence properties of the beam. Currently, also the size of the FZP is limited by the coherence length in the horizontal direction at the experiment (105 µm) (Flenner *et al.*, 2020*a* ▸). Due to the higher coherence available at PETRA IV, a larger FZP can be used than at PETRA III, resulting in a significantly higher flux at the detector. In conclusion, the upgrade to a fourth-generation synchrotron radiation source in combination with single-photon-counting detectors will open the door to even faster, dose-optimized and low-noise nanotomography, well suited for *in situ* applications.

## Figures and Tables

**Table 1:** Parameters of the TXM (Zernike phase contrast) measurements for the low-resolution and high-resolution setups

Parameter	TXM low resolution	TXM high resolution
Sample	Tardigrade	Spider hair
Energy	11 keV	11 keV
Sample–detector distance	20.4 m	20.4 m
FZP diameter	150 µm	120 µm
FZP *d*_*r*_	50 nm	30 nm
Theoretical resolution limit	30.5 nm	18.3 nm
Effective pixel size CMOS	21.4 nm	10 nm†
Effective pixel size LAMBDA	181 nm	85 nm
Magnification	303	647
Half-period resolution CMOS (3D)	98 nm	–
Scan time at resolution limit (3D)	–	60 s

† This value was calculated from the parameters and not experimentally verified.

**Table 2:** Parameters of the near-field holography measurements

Parameter	Near-field holography
Sample	Spider hair/tardigrade
Energy	11 keV
Sample–detector distance	20.6 m
FZP diameter	300 µm
FZP *d*_*r*_	50 nm
Resolution limit (focus size limited)	61 nm
Defocus distance of sample *y*_1_	62.8 mm/36.8 mm
Effective pixel size	168 nm/98 nm
Exposure time at resolution limit (2D)	300 ms

**Table 3:** Parameters of the near-field ptychography measurements

Parameter	Near-field ptychography
Sample	Spider hair/tardigrade
Energy	11 keV
Sample–detector distance	20.45 m
FZP diameter	300 µm
FZP *d*_*r*_	50 nm
Defocus distance of sample *y*_1_	36.8 mm
Resolution (2D)	177 nm/115 nm
Scan points	6 × 6
Step size (random)	1 ± 0.5 µm
Exposure time per point	1000 ms

**Table 4:** Parameters of the tomographic scans displayed in Fig. 3 ▸

Scan time (s)	No. of projections	Exposure time (ms)	Angular speed (° s^−1^)	Photons per pixel (tomogram)
6	182	10	30	1.0 × 10^4^
18	338	30	10	5.7 × 10^4^
36	682	30	5	11.5 × 10^4^
60	919	75	3	38.9 × 10^4^
180	1041	150	1	88.1 × 10^4^
360	1109	300	0.5	190.6 × 10^4^
